# Perception of having children through surrogacy in individuals with MRKH in Vietnam: a qualitative study

**DOI:** 10.3389/fpsyg.2024.1372405

**Published:** 2024-05-06

**Authors:** Thanh T. Q. Le, Nhung T. H. Le, Tuan A. Vu, Hoa H. Nguyen, Lan N. Vuong

**Affiliations:** ^1^Department of Obstetrics and Gynecology, University of Medicine and Pharmacy, Ho Chi Minh City, Vietnam; ^2^Faculty of Medicine, Department of Obstetrics and Gynecology, Vietnam National University, Ho Chi Minh City, Vietnam; ^3^Tam Gia An Psychology Clinic, Ho Chi Minh, Vietnam; ^4^Department of Laparoscopy, Tu Du Hospital, Ho Chi Minh, Vietnam

**Keywords:** Mayer-Rokitansky-Küster-Hauser syndrome, infertility, surrogacy, interview, Vietnam

## Abstract

**Introduction:**

Mayer-Rokitansky-Küster-Hauser syndrome (MRKH) is rare condition that has a negative impact on quality of life because affected women lack a uterus and vagina, and are therefore unable to engage in sexual intercourse and experience natural pregnancy. This study evaluated perceptions of surrogacy in Vietnamese women with MRKH who have started families.

**Method:**

Women with MRKH who had undergone successful vaginal reconstruction, were married, and had started families participated in a semi-structured, in-depth, one-on-one online video interview with an experienced female psychologist. Open-ended questions were used to encourage participants to express their perceptions of surrogacy; prominent themes were discussed, compared, and combined.

**Results:**

Twenty women (mean age 31 years) agreed to participate. Key themes identified from interviews were the importance of having genetic offspring, consideration of surrogacy as a preferred solution to infertility, the barriers to surrogacy in Vietnam, lack of reproductive information and counselling, individuals concealing their health condition, the impact of religion on the possibility of surrogacy, the economic cost of surrogacy, and the difficulty in finding a surrogate under the restrictions imposed by Vietnamese law.

**Discussion:**

Based on the perceptions of women from MRKH from Vietnam, there is an opportunity to improve how infertility is managed in these people, including information about surrogacy. These data show that individuals with MRKH should be provided with information about the possibility of surrogacy, encouraged to be open and seek support, and be managed by a multidisciplinary team that includes psychological support; the provision of economic support for fertility treatments in women with MRKH should also be considered.

## 1 Introduction

Mayer-Rokitansky-Küster-Hauser syndrome (MRKH), also known as congenital absence of the uterus and vagina, is a rare condition with an incidence of approximately 1 in 5, 000 live female births (Herlin et al., 2020). Although MRKH does not have a negative effect on health, it does significantly impact quality of life because affected individuals are unable to engage in sexual intercourse and experience natural pregnancy.

Vaginal reconstruction has been considered a common treatment for women with MRKH due to its simplicity and high success rate. Surrogacy and uterine transplantation are not widely performed due to high costs, the need for advanced scientific expertise, and legal complexities, and uterine transplantation only has a success rate of about 30% (Raziel et al., 2012; Arora and Blake, 2014; Zaami et al., 2017; Cabra et al., 2018; Rumpik et al., 2019; Zaami et al., 2019). Therefore, many individuals with MRKH are unable to realize their dream of becoming a mother.

The first child holds significant importance in many families, especially in cultures influenced by Eastern traditions where women face pressure regarding the obligation to continue the family lineage (Luk and Loke, 2015; Tavakol et al., 2016; Muhamad et al., 2019). It seems logical that if having a child is important and influences quality of life then surrogacy could be encouraged and considered a standard component of management strategies for individuals with MRKH. However, there is currently no research exploring the significance of having a child within the population of women with MRKH.

Surrogacy for humanitarian purposes, including in women with MRKH, has been legally permitted in Vietnam since 2015 (Vietnam Government, 2014). There are five centers in Vietnam who can facilitate surrogacy but uptake amongst people with MRKH is low. This study (NCT04923217) evaluated perceptions of surrogacy in women with MRKH who have started families. The aim was to understand perceptions regarding the importance of and motivation for having a child through surrogacy, and any obstacles preventing or delaying this type of approach to infertility. It is hoped that the information obtained will help to inform the development of appropriate policies and support strategies by policy makers and IVF centers.

## 2 Methods

### 2.1 Study design and participants

Tu Du Hospital has been providing vaginal reconstruction for women with MRKH since 2014. The research team contacted women with MRKH and invited those who had undergone successful vaginal reconstruction, were married, and had started families to participate in the study. Information about the study was provided to these individuals during an online meeting. Written informed consent was obtained from all participants before starting the interview process. Women who did not consent to having their interview recorded or who did not cooperate during the interview process were excluded. The study received ethical approval from the Biomedical Research Ethics Council of the University of Medicine and Pharmacy at Ho Chi Minh City (50/HĐĐĐ-ĐHYD), and the Scientific Research Council of Tu Du Hospital (1,351/QĐ-BVTD).

### 2.2 Interviews

Online video interviews were conducted between December 2021 and February 2022 with only the interviewer and research subject present. A semi-structured, in-depth interview method was used. The interviewer was an experienced female psychologist who created a comfortable environment for participants. Use of a semi-structured interview format allowed participants to share freely on the topic, with open-ended questions encouraging them to express their perceptions of surrogacy. Participant names were changed to a pseudonym to protect anonymity and all information was kept confidential; only the principal investigator had full access to all patient data.

### 2.3 Data analysis

Interviews were first transcribed verbatim from audio recordings, ensuring that any personally identifiable characteristics were kept confidential. Transcripts were first shown to the participants to allow them to provide comments or corrections. The transcriptions were then carefully reviewed, with important points noted by two independent researchers, then reviewed again to identify important themes. Prominent themes across all research subjects were discussed, compared, and combined. Finally, prominent themes were linked back to transcriptions to help present participants’ thoughts accurately.

## 3 Results

### 3.1 Study participants

A total of 20 women agreed to participate, of whom five had undergone previous unsuccessful surrogacy attempts (Table 1). Each interview had an average duration of approximately 25 min. Sixteen women expressed a desire to undergo surrogacy, while four did not. Reasons for not wanting surrogacy included wishing to keep their medical condition secret (*n* = 1), failed marriage (*n* = 2), or the spouse already having a child (*n* = 1).

**Table 1:** Sociodemographic characteristics of study participants.

**Characteristic**	**Range**
**Age, years**	30.9 ± 5.01 (26–41)
Marital status, *n* (%)	
Married	18 (90)
Divorced	2 (10)
Time from marriage to study period, years	3.73 ± 2.42 (0.5–8)
**Religion, *n* (%)**	
Christian	7 (35)
Buddhist	5 (25)
Ancestor worship	5 (25)
Other	3 (15)
**Socioeconomic status, *n* (%)**	
Low	1 (5)
Middle	18 (90)
High	1 (5)
Previous surrogacy attempts, *n* (%)	5 (25)
Successful surrogacy attempts, *n* (%)	0/5 (0)

Values are mean ± standard deviation (range), or number of participants (%).

### 3.2 Interview themes and comments

Paraphrased participant comments are summarized under broad themes in Table 2.

**Table 2:** Participant comments relating to different aspects of surrogacy (comments have been paraphrased).

**Significance of genetic offspring**	**Surrogacy as a preferred solution**	**Lack of reproductive information and counselling**	**Economic considerations**	**Impact of religion**	**Difficulty finding a surrogate**
Until I started a family, the pain and longing for a child kept urging me, and I explored surrogacy	In 2016, I returned to the hospital because at that time, the law allowed surrogacy, and I also had hope	I did not listen carefully when the doctor said my ovaries were normal. So when I apply for surrogacy, I will use my sister’s eggs	I know people who have a normal uterus but cannot conceive, and they use IVF. I see that the success rate is not high, and it’s also expensive. Therefore, sometimes, I also think about whether to adopt a child	I am Catholic. In the faith, IVF goes against God’s will, so people see it as a sin. So, I dare not use this IVF method	In Vietnam, the surrogate needs to be a close relative and I have not found such a person so it’s difficult
I’m afraid that if we do not have children, the marital relationship will not be sustainable	I have researched surrogacy, and I see that many cases are successful, so I’m not overly worried	Only when I had surgery did I know that I could not give birth. Living together, not matching each other, and with the issue of children, we broke up	Whenever there is enough economic capacity, I will start going to the hospital for assistance	For cases like mine, with a congenital disease, I hope that surrogacy is considered permissible. We are born with the condition, so we hope that those who undergo artificial processes are not considered guilty	Even though we have an embryo, we have not yet found a surrogate who meets the requirements
I am under pressure from my in-laws regarding the issue of children	My husband said if natural conception does not work, then we can adopt, but my husband still prefers a child born with our shared genes		I have 5 siblings and had to help with their college education so I cannot do both things at the same time. Now, I have saved enough money for surrogacy, but I am old	The faith knows that it’s not allowed, but now there’s only this way left. So, if I want it, I must accept it; I do not know what to do now	I will donate eggs again so that we have more eggs and embryos to use if we can find a surrogate; however, my age and the quality of my eggs mean that we cannot wait for a long time
Being a wife is not enough; being a mother is not enough					Although my sister volunteered to be a surrogate, it would be hard for her and could make her husband angry that she has my child, and I do not want to put her through that
No one knows everything, so sometimes people ask if I have a baby. After that, I feel a bit sad, like I do not know how to respond. I will try to change the subject instead of saying that I am ill					

IVF, in vitro fertilization.

#### 3.2.1 Significance of having genetic offspring

After undergoing vaginal reconstruction, most women with MRKH considered having children as their greatest desire in life. This intense desire for offspring was described by one respondent as “pain and longing.” Many individuals perceived that not having shared children can strain marital relationships, and felt pressure and criticism from their husband’s family regarding their inability to conceive. The presence of step-children was something that reduced the motivation to undergo surrogacy for one respondent, who preferred to nurture family happiness. For others, the end of their marriage removed the motivation to consider surrogacy. Two individuals in the study population were divorced, and inability to have a genetic offspring was a contributor to the marriage breakdown (alongside incompatibility and an unfaithful partner).

#### 3.2.2 Surrogacy as a preferred solution

When interviewed about childbirth, most participants stated a preference for having their own child rather than adopting a child. This suggests that these individuals prioritize exploring surrogacy to have a genetically related child, and they were glad that surrogacy was now permitted in Vietnam. The ability to easily access information online and plan for surrogacy made it somewhat easier for couples looking into this option. Couples expressed desire to have a child that is genetically related to both parents, influencing the choice of most appropriate infertility treatment.

#### 3.2.3 Barriers to surrogacy in Vietnam

Despite the strong desire for children, women with MRKH stated that they face numerous difficulties in pursuing surrogacy in Vietnam, as described in more detail in the paragraphs below. Therefore, while the desire to have a child via surrogacy might be strong, this has not yet been achieved for most.

#### 3.2.4 Lack of reproductive information and counselling

Most information about surrogacy is self-researched online, but there is a lack of information about MRKH syndrome or reproductive capabilities. Many participants have misconceptions about their reproductive abilities, and some did not realize that they could use their own eggs for surrogacy. Others were not completely aware that surrogacy was an option for them.

#### 3.2.5 Concealment of medical conditions and fear of discovery

Most respondents stated that they keep their medical condition a secret and felt pressure when others enquire about their childbearing status. Some even conceal their medical conditions from their spouses due to feelings of shame. They worry that pursuing surrogacy may reveal their secret, affecting marital happiness. One, who declined to have a child, hides her condition by claiming she’s not ready to give birth.

#### 3.2.6 Economic considerations

High treatment costs with low success rates impact the decision to pursue surrogacy for some women. They see the cost of *in vitro* fertilization (IVF) and feel that surrogacy would only be an option if they were financially stable. Family financial commitments, such as schooling for younger siblings, was another financial barrier to surrogacy meaning that some had to postpone their dream of having a child.

#### 3.2.7 Impact of religion

In Catholicism, reproduction must follow natural processes, preventing individuals of this faith from pursuing surrogacy. Others considered that the fact that MRKH is an innate condition might mean that they could undergo infertility treatment such as surrogacy without being considered “guilty” by the church. Another respondent felt that they had no other option but surrogacy even though it was forbidden by the church.

#### 3.2.8 Difficulty finding a surrogate

Many participants mentioned challenges in finding a surrogate who meets the legal requirements in Vietnam. Even if they do find one, there may be no other options if the first fails. Furthermore, the issue of the potential negative impact of being a surrogate on a family member was an issue for some participants.

## 4 Discussion

The results of our survey indicate that women with MRKH have a strong desire for genetic offspring. Study participants often used terms such as “aspiration,” “desire,” and “obsession” to describe their longing for a biological child.

In Vietnamese society, influenced by Eastern culture, the motivation to have children arises not only from internal desires but also from external pressures, including marriage, family, and society. Most study participants felt that having children was essential for sustaining a marriage. The inability to have children can lead to stress, marital unhappiness, and even divorce; two of the 20 participants in this study experienced marital breakdown that were in part due to child-related issues. A Chinese study of women with infertility showed that infertile couples had double the divorce rate of non-infertile couples (Che and Cleland, 2002). Research on infertile women in Eastern cultural countries such as China, Malaysia, and Iran has also highlighted the societal pressure on infertile couples, especially on women who are expected to maintain the family lineage for their husband’s family (Luk and Loke, 2015; Yazdani et al., 2016; Hibino, 2019; Tavakol et al., 2019). In our study, women with MRKH expressed significant pressure from their husband’s parents when unable to fulfill this obligation, and they felt judged by society for not having children. These societal and familial pressures can have a profound impact on the mental well-being and social relationships of women with MRKH, motivating them to seek options that allow them to have genetic offspring.

Today, reproductive options for women with MRKH include IVF with surrogacy, uterine transplantation, or adoption if the other methods are not feasible (Brännström et al., 2014; Friedler et al., 2016; Testa et al., 2018; Peters et al., 2020). Uterine transplantation is a less-established method with potential risks for both mother and baby (Friedler et al., 2016). Surrogacy appears to be a good option for women with MRKH, particularly in countries where this is permitted (Fedele et al., 2021). In Vietnam, surrogacy for humanitarian purposes has been allowed since 2015. The current law (Vietnam Government, 2014, 2015, 2016) allows altruistic gestational surrogacy for couples with infertility where the surrogate needs to be a full sibling, half sibling, sibling-in-law or full cousin of one member of the affected couple. Surrogates can only do this one (i.e., cannot have previously carried a surrogate child) and must have given birth at least once before. To be eligible for surrogacy, couples must meet medical eligibility requirements, have had at least 1 year of IVF and provide a written dossier to apply for approval to undergo surrogacy. There are also requirements for health, psychological and legal counselling before surrogacy can be approved. This is a multidisciplinary and complex process that needs to take the specific needs of each couple and their potential surrogate into account. In Vietnam, law mandates the involvement of a psychologist to provide counselling to both the infertile woman and the surrogate (Vietnam Government, 2015).

Most women with MRKH perceive surrogacy to be the only method that allows them to have genetically-related children. However, only 25% of the participants in our study had taken this option, and 10% were not even aware that surrogacy was a possibility for them. Some participants mentioned unhelpful information from healthcare professionals as contributing to misconceptions and emotional distress. Many women with MRKH only seek medical attention due to the absence of a vagina, lack of menstruation, and/or inability to engage in sexual intercourse. At this stage, doctors focus on creating a vaginal structure and often neglect to advise on fertility options. Most information about surrogacy is obtained through self-research on the internet, which may lead to misunderstanding and delayed treatment decisions. Therefore, providing information about surrogacy at healthcare facilities who provide care for women with MRKH who seek medical attention is crucial.

Another challenge is that women with MRKH often conceal the fact that they lack a vagina and uterus and are concerned that the process of surrogacy may reveal this, causing emotional distress. Despite the benefits of sharing medical conditions, many women with MRKH still struggle to overcome the fear of rejection, lack of empathy, and fear of being perceived differently (Ernst et al., 2016). Surrogacy is a challenging and demanding process and, to succeed, patients require substantial support from their husbands, families, and those around them. Supporting patients in overcoming their fear and self-esteem issues, sharing their condition with others for assistance, is essential. Choosing the right time and appropriate individuals to share their experience with is crucial to receive support and avoid uncomfortable situations or rejection (Holt and Slade, 2003).

The current average income in Vietnam is around 7 million VND per month (approximately 286 USD). The cost of surrogacy ranges from 2,500 to 12,500 USD, making it very high compared with the average income (CNY Fertility, 2023; Take-Profit.org, 2023). Since the implementation of surrogacy services in 2016, data from Tu Du Hospital indicate that only 25% of the 20 cases of surrogacy (including 7 cases with congenital uterine absence) resulted in successful births (Chau, 2016). In our study, most participants only had enough income to cover their living expenses, and only five had pursued surrogacy, with no reported successful cases. There is therefore the opportunity to raise awareness at a policy level to improve options for women with MRKH, including the provision of funding for infertility treatment.

The main religion in Vietnam is Buddhism, which has no specific restrictions or judgment about infertility and the inability to have a child. In contrast, for Catholics, Canon law prohibits the use of assisted reproductive methods, including IVF and surrogacy (Schenker, 2005). More than one-third of the participants in our study were Catholic, and overall nearly 10% of the Vietnamese population is Catholic. The Catholic Church in Vietnam follows the Canon law regulations, creating a barrier to surrogacy for Catholic women with MRKH.

A key reason why women with MRKH in this study had not pursued surrogacy was the difficulty in finding a suitable surrogate within the Vietnamese legal framework (Vietnam Government, 2015, 2016). According to the Marriage and Family Law (No. 52/2014/QH13). Criteria for a humanitarian surrogate include being a close relative of the wife or husband, having given birth before and only being allowed to be a surrogate once, being of an appropriate age with confirmation of reproductive ability by a qualified healthcare organization, obtaining the written consent of the surrogate’s husband if they are married, and receiving counseling on health, legal, and psychological aspects (Vietnam Government, 2014, 2015, 2016). In addition to these specific local criteria, there are also numerous health conditions that influence fertility and could prevent relatives from being able to act as a surrogate for infertile family members (Marinelli et al., 2022b; Bayoumi et al., 2024; Choi, 2024). Participants in our study mentioned the considerable challenge of finding a suitable relative to act as a surrogate, the burdensome childbirth process, and the difficulty of adhering to the legal criteria. Research on non-commercial surrogacy in Vietnam also emphasized the significant challenge of finding a surrogate within the legal framework and noted that the act of carrying a pregnancy for 9 months, giving birth, and handing the child to another family can cause severe psychological and physical trauma to the surrogate (Hibino, 2019). Furthermore, the Vietnam government’s encouragement to limit families to two children reduces the availability of close relatives willing to act as surrogates for the current generation.

For women with MRKH, new technologies may provide an alternative to surrogacy and allow them to achieve genetic offspring. These include the potential for a bioengineered uterus created using tissue engineering principles (Zaami et al., 2019). Time will tell whether new techniques and technologies have the potential to contribute to satisfactory solutions to infertility for couples where the female partner has MRKH.

This is the first study assessing the perception, motivation, and barriers to surrogacy in women with MRKH from Vietnam. The interview process used allowed participants to express their thoughts and experiences, providing authentic and in-depth information. However, our sample size is small, with only 20 cases, but MRKH is rare meaning that a large sample size was never going to be possible to recruit a larger patient population (especially from a single center). Therefore, we believe that the chosen qualitative analysis method and sample size are suitable for this type of research. For a broader picture of MRKH in Vietnam, it would have been useful to know the number of affected individuals and the proportion who had undergone successful vaginal reconstruction. However, we only have information for women with MRKH who came to our hospital for vaginal reconstruction.

In conclusion, the information obtained highlights the importance of having children through surrogacy for individuals with MRKH after starting a family, and the challenges they face in seeking support. The issues reported by women with MRKH in Vietnam who wish to have genetic offspring in the current study exist in the broader context of the numerous complexities, and the psychosocial, ethical and legal issues that surround surrogacy (Saxena et al., 2012; Burrell and O’Connor, 2013; Blazier and Janssens, 2020; Igareda González, 2020; Swanson et al., 2020; Frati et al., 2021; Marinelli et al., 2022a). These include balancing the rights and medical/psychological wellbeing of the prospective mother, the surrogate and the child. Therefore, it would be desirable to develop guidelines to regulate surrogacy (and international surrogacy) worldwide based on a set of common norms, sharing core values, according to principles that support the best interests of the child, and consistent with the right to family life. Despite the many challenges, it would be inappropriate to ignore the value that surrogacy can have for the countless women with MRKH. Therefore, surrogacy should be considered and viewed as an appropriate option in women with MRKH. Based on the responses from participants in our study, we suggest that individuals with MRKH should be provided with information about the possibility of surrogacy, encouraged to be open and seek support, and managed by a multidisciplinary team that includes psychological support. In addition, there is a need for economic support policies, such as health insurance, to assist with the costs of fertility treatments for individuals with congenital lack of a uterus.

## Data availability statement

The raw data supporting the conclusions of this article will be made available by the authors, without undue reservation.

## Ethics statement

The studies involving humans were approved by the Biomedical Research Ethics Council of the University of Medicine and Pharmacy at Ho Chi Minh City and the Scientific Research Council of Tu Du Hospital. The studies were conducted in accordance with the local legislation and institutional requirements. The participants provided their written informed consent to participate in this study.

## Author contributions

TL: Conceptualization, Data curation, Formal analysis, Methodology, Project administration, Writing – original draft, Writing – review & editing. NL: Conceptualization, Data curation, Methodology, Writing – review & editing. TV: Data curation, Writing – review & editing. HN: Conceptualization, Methodology, Supervision, Writing – review & editing. LV: Conceptualization, Methodology, Supervision, Writing – original draft, Writing – review & editing.
